# The Applications of Sensors and Biosensors in Investigating Drugs, Foods, and Nutraceuticals

**DOI:** 10.3390/s19153395

**Published:** 2019-08-02

**Authors:** Luigi Campanella, Mauro Tomassetti

**Affiliations:** Department of Chemistry, “La Sapienza” University of Rome, 00185 Rome, Italy

**Keywords:** analysis, food, drugs, clostridium, ethylvanillin, antioxidant properties, ethanol in pharmaceuticals by DMFC, electronic nose and tongue, intelligent food packaging

## Abstract

The present Special Issue is focused on developing and applying several sensors, biosensor devices, and actuators for the analysis of drugs, foods, and nutraceuticals. Some applications concern classical topics, such as clostridium determination in dairy products, flavouring material in foods like ethylvanillin, or the antioxidant properties of fruit juices, while other applications are more innovative, such as food safety analysis, artificial human senses (electronic nose, or tongue) development, or ethanol determination in pharmaceutical drugs, or forensic purposes using catalytic fuel cell; and lastly, new studies devoted to intelligent food packaging. Therefore, this Special Issue should interest both specialists in the sector and readers who are simply curious, or are simply interested in innovations in the field of food and drug analysis.

## 1. Introduction

The development of sensors and biosensors in the last period is mainly due to two reasons, one of technical origin, the other of a social one. The needed control to ensure safety and security for citizens was for many years based on separation techniques, mainly chromatographic techniques coupled with extremely sensitive detection methods, starting with mass spectrometry. This approach is generally expensive, time consuming, and labour intensive, so that rapid and cheap alternatives are looked for. On the other hand, we have increasing attention and interest paid to the safety of foods and nutraceuticals (these ones continuously more used than drugs) from the civil society, because of a common feeling to have a safer life.

## 2. Contributions

This issue of SENSOR wishes to contribute to reporting the advancements and progress of the scientific research applied to the design and setup of sensors and biosensors in investigating drugs, nutraceuticals, and foods. The issue contains seven papers moving across different aspects of the central theme.

In the first paper, Chinese researchers (Qian et al.) [1] describe a sensoristic method to determine Clostridium perfringens in dairy products by a very innovative biosensor, based on DNA-modulated properties of CeO_2_ nanorods. Clostridia are anaerobic bacteria possibly present in the human intestine, even of newborns, but also in animals, soil, and decomposing plants. They are responsible for many diseases and pathologies as, if assumed with foods, they are multiplied, producing a dangerous toxin.

The second paper [2] describes a preliminary study about intelligent food packaging. Packaging is the way to make available any kind of product in the space and in the time. Between the container and the contained material, symbiosis must be established by which a new subject arises, able to protect the product from chemical and physical stresses and to emphasize its characteristics. All this is particularly important in the case of foods to be consumed by humans in the future.

The third paper by Mao et al. [3] faces a completely new theme related to the last development of artificial human senses, starting with the electronic nose and tongue. In particular, the proposed sensor is a broad-spectrum sweet-tasting one, based on the Ni(OH)_2_/Ni electrode couple. Humans can distinguish between five basic tastes, including sweet, salty, umami, bitter, and sour. Recently, lipid sensors have been identified on the tongue, which suggest that fat can be considered the sixth taste. In this paper, the sweet taste is considered. The taste receptor is a type of receptor that facilitates the sensation of taste. When food or other substances enter the mouth, molecules interact with saliva and are bound to taste receptors in the oral cavity and other locations.

The fourth paper by Gupta et al. [4] deals with the determination of vanillin in foods. Because of its aromatic properties, vanillin, the main aromatic compound in natural vanilla, is the most widely used flavouring material in foods and beverages. On the other hand, ethylvanillin, which is commonly used as another synthetic compound, has much more flavouring strength than vanillin and is used in the formulation of imitation products. Thus, methods able to determine vanillin are particularly requested in order to discover such imitations.

The fifth paper is from an Italian team and reports a study in which a direct methanol fuel cell is proposed to find ethanol in several pharmaceuticals and forensic samples [5]. The paper refers, extending its application, to an alcoholmeter, where breath samples are received and, due to the catalytic properties of the electrode material, are transduced into an electric current, the intensity of which is proportional to the alcohol concentration in the breath sample.

The sixth paper is central to the issue’s aim, being strictly related to food safety analysis and expressively performed in points of care (POC) where the traditional chromatography–mass spectrometry hyphenated method is impeded due to its characteristics of requesting a long time and expert hands to get the results [6]. Food safety issues have recently attracted public concern. The deleterious effects of compromised food safety on health have rendered food safety analysis an approach of paramount importance. There is therefore an urgent need to develop simple and robust diagnostic POC devices, typically rapid, cost effective, and user friendly, and able to detect food contaminants, such as foodborne pathogens, chemicals, allergens, and toxins.

Fruits and derived products, such as fruit juices, are a significant source of many biologically active antioxidant compounds, such as ascorbic acid, tocopherols, carotenoids, and polyphenols. These antioxidants found in fruit juices, such as pomegranate, apple, orange, guava, peach, and other mixed fruits, are thought to contribute towards positive health outcomes in cases of cardiovascular disease, cancer, and degeneration, reasons why their consumption in the last few years has increased at a very quick rate. Consequently, the interest to design and set up new analytical methods is worldwide. The paper by Elbehery tries to give a new answer to this question [7].

In summary, the issue succeeds in giving a strong, accurate, and innovative picture of the research in sensor science, applied to foods and nutraceuticals, and also extending to drugs, as interested matrices to the same common problems: Safety, quality, and packaging.
